# Terminal Crossbreeding of Murciano-Granadina Goats to Boer Bucks: Effects on Reproductive Performance of Goats and Growth of Kids in Artificial Rearing

**DOI:** 10.3390/ani11040986

**Published:** 2021-04-01

**Authors:** Ion Pérez-Baena, Marta Jarque-Durán, Ernesto A. Gómez, José-Ramón Díaz, Cristòfol Peris

**Affiliations:** 1 Institut de Ciència i Tecnologia Animal, Universitat Politècnica de València, Camí de Vera s/n, Valencian Community, 46022 València, Spain; iopebae@upv.es (I.P.-B.); marjardu@alumni.upv.es (M.J.-D.); 2 Centro de Investigación y Tecnología Animal, Instituto Valenciano de Investigaciones Agrarias, Apartado 187, Castellón, Valencian Community, 12400 Segorbe, Spain; gomez_ern@gva.es; 3 Departamento de Tecnología Agroalimentaria, Escuela Politècnica Superior de Orihuela, Universidad Miguel Hernández, Ctra. Beniel, Km 3.2, Alicante, Valencian Community, 03312 Orihuela, Spain; jr.diaz@umh.es

**Keywords:** goats, kids, Murciano-Granadina, Boer, reproductive performance, growth

## Abstract

**Simple Summary:**

The Murciano-Granadina is one of the main goat breeds dedicated to milk production worldwide. However, kids of this breed are born with low birth weight and do not grow rapidly, so farmers have little interest in raising them for meat because of the low profitability they obtain. The aim of this work is to improve the growth characteristics of these kids by crossbreeding Murciano-Granadina goats with males of the Boer breed, which is considered the best meat goat breed. The results showed that crossbred kids have a higher birth weight (+24%) and, in artificial rearing until 9 kg live weight, have lower mortality (−37%), higher growth rate (+32%) and a better milk powder conversion rate (16%). However, when this crossbreeding is carried out in the anoestrous season (experiments conducted at latitude 38–39° N), the reproductive performance of Murciano-Granadina females worsens slightly. It is concluded that the terminal crossbreeding of Murciano-Granadina goats to Boer bucks (those not used to produce replacement kids) could be an interesting option for ethical goat production.

**Abstract:**

The aim of this work was to improve the growth characteristics of Murciano-Granadina (MG) kids through terminal crossbreeding of MG goats to Boer bucks. Four experiments were carried out, using a total of 354 MG goats, half of which were mated with MG bucks (n = 12) and the other half with Boer bucks (n = 12). The kids were raised in artificial rearing until slaughter weight (9 kg). The birth weight and average daily gain were recorded in crossed kids (n = 197 and 145, respectively) and purebred kids (n = 257 and 169, respectively). Crossed kids presented significant differences (*p* < 0.001) compared to MG purebred kids in birth weight (+ 24%), mortality in artificial rearing (−37%), average daily gain (+32%) and milk powder conversion rate (−16%). However, the reproductive performance rates of MG goats mated with Boer bucks were slightly worse (pregnancy rate: 78.5% vs. 86.6%, *p* < 0.05; kidding rate: 62.0% vs. 75.7%; *p* < 0.01; prolificacy: 1.9 vs. 2.1 kids/parturition), especially when the matings took place in non-breeding season (experiments conducted at latitude 38–39° N). It is concluded that the terminal crossbreeding of MG goats to Boer bucks (those not used to produce replacement kids) could be an interesting option for ethical goat production.

## 1. Introduction

In Spain, several native dairy goat breeds are used, among which the Murciano-Granadina (MG) breed stands out due to its higher census (estimated at about 500,000 animals [1]) and international diffusion (found in Mediterranean and Latin American countries and Western Africa). Goats of this breed have good milk production and quality records (average of the official milk recording in 2020: 547 kg in 210 days of lactation; 5.47% fat and 3.52% protein [2]) and are well adapted to semi-intensive and intensive production systems and to being machine-milked once a day [3]. From a reproductive standpoint, the MG breed does not present a deep seasonal anoestrus. Thus, in the spring months in the temperate latitudes of the southern Iberian Peninsula (36–38° N), the males maintain a high libido and the MG does are completely responsive to the male effect, without artificial manipulation of the photoperiod [4]. Females present high fertility (usually around 80–90% in females naturally mated during breeding season) and prolificacy (herd averages around 1.8 kids/parturition) [5]. However, a weak aspect of the MG breed is that kids have a low growth rate, generally between 100 and 140 g/day in artificial rearing [6,7,8]. This could be explained by the limited mature size of the breed (males: 60–70 kg; females: 45–55 kg) [5], as growth rate in goats is dependent on mature size of a particular breed [9]. In Spain, at present, MG kids not destined for replacement are usually slaughtered for meat at a very young age, such as milk-fed kids at 35–45 days of age and a live weight of 7–9 kg. Kid meat is a product highly appreciated by consumers in the Spanish goat milk production regions, and the peaks of demand are mainly during the Christmas and Easter holidays. Despite this, farmers tend to consider MG kids as a byproduct and have little interest in raising them for meat, given the low profitability they obtain. This situation is undesirable, as it could lead to the emergence of practices that are incompatible with ethical goat production. By analogy to what happens in other species and goat breeds, the fastest way to improve the growth characteristics of kids and, consequently, to increase the profitability of their breeding for meat, would be the use of a terminal cross with males belonging to a breed with meat aptitude and high growth rate [10]. This mating would apply only to MG goats that are not going to be the dams of replacement animals, and all crossbred kids should be slaughtered for meat production when they reach the appropriate weight for sale. However, it is surprising that such a cross has never been studied in the Murciano-Granadina breed.

The Boer goat is a meat aptitude breed of South African origin that has spread all over the world to be exploited as a pure breed (body mature weight: 70–80 kg female, 100–120 kg male [11]), or for terminal crossbreeding with native goat breeds to improve growth and/or carcass quality of kids [10]. According to the literature, the animals of this breed are fast growers (>200 g/d), hardy and adaptable, disease-resistant, fertile and produce meat of high quality [11,12,13,14]. In crossbreeding, it has been shown to improve the growth of local breeds in some countries, such as: USA (Spanish breed [15,16]), Mexico (Creole breed [17]), Poland (White Improved breed [18]), Brazil (local goats [19]) or China (Chengdu Ma breed [20]).

On the basis of the cited precedents, we proposed to study the terminal crossbreeding of meat-suitable Boer breed males with MG females in experiments performed on two experimental farms. The aim of this paper is to study the reproductive traits of the dams and the growth of the crossbred kids in artificial rearing, also considering other factors that, according to the literature, could affect the variables mentioned above (reproductive traits: parity number of goats and season [21]; growth traits: litter size and sex of kids [6,22]). Our hypothesis is that this terminal crossbreeding improves the growth traits of the MG kids, without a negative effect on the reproductive traits of MG goats.

## 2. Materials and Methods

### 2.1. Experimental Design

Four experiments were carried out using a total of 354 MG goats (106 nulliparous and 248 lactating goats), half of which were mated with MG bucks (n = 12) and the other half with Boer bucks (n = 12; Table 1). Experiments 1 and 2 were carried out at the experimental farm of the Universitat Politècnica de València (39°28′ N; 0°22′ E) and matings took place in February–March (non-breeding season). Experiments 3 and 4 were carried out at the experimental farm of the Universidad Miguel Hernández in Orihuela (38°05′ N; 0°56′ E), and matings took place in September–October (breeding season; experiment 3) and February–March (experiment 4). Both experimental farms are located in the Valencian Community (Spain) and the annual health checks performed by veterinary services showed that they were free from brucellosis, tuberculosis, *Mycoplasma agalactiae* and caprine arthritis-encephalitis virus.

In each experiment the mating groups were organised in the same way: (a) nulliparous goats were separated into two homogeneous groups in terms of weight and age, and each group was randomly assigned to mating with Boer bucks (group 1) or MG bucks (group 2); (b) lactating goats were also separated into two homogeneous groups in terms of parity, live weight and milk production, and each group was randomly assigned to mating with Boer bucks (group 3) or MG bucks (group 4). Each group was located in different pens and no hormonal treatment was applied to either the bucks or the goats. Table 2 summarises the number of bucks and goats used in each of the four groups mentioned, for each experiment.

For each experiment, all the bucks were introduced to the mating groups on the same day, and they were in contact with the females for a total period of 40 days. At 70–75 days after introducing the bucks to the goats, pregnancy was determined by transabdominal ultrasound (5.0 MHz). Abortions, stillbirths, kiddings and litter size were recorded. All the kids were weighed at birth and raised artificially.

A total of 314 kids from single, double and triple parturitions were used for the growth study (145 BO × MG and 169 MG × MG; Table 1). In experiment 1, kids were housed in pens with 2 or 3 animals grouped according to genotype (crossbred or purebred), sex and birth weight and were fed in artificial rearing in buckets; in 17 pens (8 with MG kids and 9 with BO × MG kids) the consumption of powdered milk was recorded daily to calculate the conversion rate. In the rest of the experiments, kids were reared with automatic suckling machines in separate groups according to genotype. All the kids that participated in the growth study were weighed twice a week until 9 kg live weight (slaughter weight).

### 2.2. General Management

Does of experimental groups were kept in indoor pens (size = 1.5 m^2^/goat; feeder = 0.5 m/goat; 3-bowl water troughs per pen) and received a mixed ration twice daily (at 0900 and 1800 h) throughout the experimental period. The ration was formulated according to [23] for goats in lactation and gestation using lucerne hay, straw and commercial feed. One month before kidding, does were dewormed and vaccinated (*Clostridium perfringens* Types C and D plus *Corynebacterium pseudotuberculosis* and tetanus toxoid). Following birth, kids received AD_3_E·vitamin (1 mL/kid; Vit AD_3_E oral, Industrial Veterinaria S.A., Esplugues de Llobregat-Barcelona, Spain) and were ear-tagged, separated from does and bottle-fed with colostrum for the first 48 h of life. Experimental kids were ad libitum artificially reared with warm (36–38 °C) commercial milk replacer (experiment 1: Nantamilk Supreme, Nanta S.A., Madrid, Spain; experiments 2, 3 and 4: Agno Chevro 63, Celtilait, Ploudaniel, France) reconstituted at 17% (*w*/*w*). In the first days, kids were trained to suckle from an artificial nipple. Kids were incorporated into the experiments when it was confirmed that they could suckle directly from the nipples. In experiment 1, the reconstituted milk replacer was offered twice daily in containers fitted with teats, and kids were reared in straw-bedded pens (size: at least 0.3 m^2^/kid). In the other experiments, milk was supplied with automatic suckling machines (JR AG-21, Industrias JR, Valdelafuente, Spain) and the kids were housed in artificial rearing slatted-floored rooms during the first 20 days of age, and thereafter were reared in straw-bedded pens (0.3 m^2^/kid).

### 2.3. Measured Variables

Three components of reproductive performance were considered: pregnancy rate (as %: number of pregnant does × 100/number of does exposed to bucks); kidding rate (as %: number of does kidding × 100/number of does exposed to bucks) and prolificacy (number of kids born/number of does kidding). Abortions, goat mortality, stillbirths and kid mortality during rearing were also recorded.

Kids were weighed using a digital dynamometer (KERN^®^ HDB 10K10, readability ±10 g, Masnou, Spain) at birth and every 3–4 days (twice a week) thereafter until reaching slaughter weight (9 kg live weight). For each kid, average daily gain (ADG) and age to reach 9 kg live weight were calculated.

In experiment 1, daily milk replacer intake of each pen was determined by weighing milk supplied and milk refusals. A 50 mL sample of milk refusals was taken daily from each pen to determine dry matter, according to [24]. This way, the daily dry matter intake of milk replacer was calculated for each pen. Feed conversion ratio for milk replacer was calculated for each pen as kg of dry matter (DM) milk replacer ingested/kg live weight gain. In the other experiments, the total weight of powdered milk delivered with the automatic suckling machines was recorded for each of the experimental groups and feed conversion ratio for milk replacer was calculated globally for each experimental group (crossed kids and MG purebred kids). However, in experiments 2 and 4, it was not possible to calculate the milk powder conversion rate due to accidents that caused losses of reconstituted milk during breeding (disconnection of the nipples).

### 2.4. Statistical Analysis

Binary variables (pregnancy rate, kidding rate, abortion rates, goat mortality and kid mortality) and ordinal variables (prolificacy: 1, 2 ≥ 3 kids/parturition) were analysed applying logistic regression models with the Logistic procedure of the SAS package [25], considering the following factors: buck breed (Boer vs. MG), parity (nulliparous vs. lactating goats), experiment (1 to 4) and interactions (buck breed × parity and buck breed × experiment). Regression coefficients from the logistic regression were exponentiated to obtain the odds ratios and corresponding 95% confidence interval associated with each factor. For dichotomous factors (buck breed and parity), an odds ratio significantly higher (or lower) than 1 indicated an increased (or reduced) risk of pregnancy (or other binary variables) if the factor was present. For class factors (experiment), one class of each factor was considered as the reference, and an odds ratio significantly higher (or lower) than 1 for any other class of this factor was indicative of an increased (or reduced) risk of pregnancy (or the other binary variables) when compared to the reference class. For the prolificacy variable, an ordinal logistic regression model was used (proportional odds model).

Quantitative variables recorded in the kids (birth weight, ADG and age up to 9 kg live weight) were statistically analysed using the GLM procedure from the SAS package, considering the following factors: genotype (MG purebred vs. BO × MG) and sex (male vs. female) of the kids, litter size (single, twin and triplets), parity of the goats (1 and ≥2), experiment (1, 2, 3 and 4), the double interactions genotype × sex, genotype × litter size, genotype × parity and genotype × experiment and the triple interaction genotype × sex × litter size. With the effects that were significant (with more than two levels), pairwise comparison of means were made applying Student *t*-test. Finally, the consumption and the conversion ratio of powdered milk of 17 pens in experiment 1 (4 and 4 pens for MG male and female kids, respectively; 5 and 4 pens for BO × MG male and female kids, respectively) were analysed with the GLM procedure from the SAS package, considering the fixed effects of genotype (MG purebred vs. BO × MG), sex (males and females) and the genotype × sex interaction.

## 3. Results

### 3.1. Goats’ Reproductive Performance

Ten goats, five mated with Boer bucks and five mated with MG bucks, died before verification of whether or not they were in gestation and were therefore not considered in the pregnancy rate.

The average pregnancy rate was 82.6% and it was affected (Table 3) by the buck breed (*p* < 0.05), parity (*p* < 0.05) and experiment (*p* < 0.05). The average kidding rate was 68.9% and the effects of the buck breed (*p* < 0.01), parity (*p* < 0.01) and experiment (*p* < 0.01) were also significant (Table 4). Overall, goats mated with MG bucks had a higher pregnancy rate (86.6%) and kidding rate (75.7%) than goats mated with Boer bucks (78.5% and 62.0%, respectively); the corresponding odds ratios (OR) were 1.81 (Table 3) and 1.99 (Table 4), respectively. Values of pregnancy rate in the experiment carried out in breeding season (experiment 3) were 88.3% and 86.7% for goats mated with MG and Boer bucks, respectively; the values in the experiments performed in non-breeding season were: 92.6% and 70.4% for experiment 1, 89.1% and 84.4% for experiment 2 and 79.5% and 62.5% for experiment 4. Kidding rate values for each experiment were: 72.6% and 67.7% for experiment 3, 88.9% and 63.0% for experiment 1, 82.6% and 71.7% for experiment 2, 64.3% and 45.2% for experiment 4. However, the buck breed × parity interaction was not significant for either of the two variables (*p* > 0.05). Primiparous goats had a better pregnancy rate and kidding rate than multiparous goats (OR = 2.31 and 2.68, respectively). With respect to the experiment factor, we can highlight that experiment 4 had the lowest values for pregnancy rate (70.9%) and kidding rate (54.8%), presenting significant differences compared to experiments 2 (*p* < 0.01) and 3 (*p* < 0.01) for pregnancy rate and experiments 1 (*p* < 0.01), 2 (*p* < 0.01) and 3 (*p* < 0.05) for kidding rate.

The mean values for abortions and mortality rates of goats in the four experiments were 4.5% (16 of 354 goats) and 8.7% (31 of 354 goats), respectively, and were not significantly affected by any of the factors considered in the statistical model.

Goats mated with Boer bucks had a prolificacy of 1.9 ± 0.68 (m ± SD) kids/parturition, with a frequency of singles, twins, triplets and quadruplets of 30.3%, 55.0%, 13.8% and 0.9%, respectively. The corresponding values for goats mated with MG bucks were 2.1 ± 0.75 and 20.9%, 56.7%, 19.4% and 3%. Statistical analysis (Table 5) shows that there was a higher probability of increasing prolificacy when using MG bucks, compared to Boer bucks (OR: 1.98; *p* < 0.01) and in multiparous goats, compared to primiparous goats (OR: 6.55; *p* < 0.001). The prolificacy in the experiment conducted in breeding season (experiment 3) was 2.08 ± 0.72 and 2.07 ± 0.73 for goats mated with MG and Boer bucks, respectively; the corresponding values for the experiments carried out in non-breeding season were 1.88 ± 0.54 and 1.59 ± 0.62 for experiment 1, 2.29 ± 0.77 and 1.91 ± 0.58 for experiment 2, 1.85 ± 0.86 and 1.53 ± 0.61 for experiment 4. The experiment factor also significantly affected (*p* < 0.001) prolificacy, as it was higher in experiments 2 and 3 compared to experiment 4 (OR = 3.83 and 4.75, respectively; *p* < 0.001 in both cases). In contrast, prolificacy did not differ significantly between experiments 1 and 4 (*p* = 0.31). Interactions were not significant.

### 3.2. Kids: Birth Weight, Growth and Mortality

The birth weight of the kids was significantly affected by the following factors: genotype (*p* < 0.001), sex (*p* < 0.001), litter size (*p* < 0.001), experiment (*p* < 0.001) and genotype × sex interaction (*p* = 0.036). The genotype × experiment interaction and the other factors considered in the model were not significant (*p* > 0.05). On average, BO × MG crossbred kids weighed 0.54 kg more at birth than MG kids (Table 6), representing an average increase of 24%. In addition, this higher weight of the crossbred kids compared to the MG purebred kids was observed both in males and females, and in single, double and triple birth. The fact that the genotype × sex interaction was significant may be explained by the fact that the differences in birth weight between the two genotypes were greater in males (0.64 kg) than in females (0.44 kg), but in the two cases differences were significant (*p* < 0.01; Table 7). For the other significant factors, the following results were obtained (Table 6): (a) male kids had a higher birth weight than female kids; (b) single parturition kids weighed more than those from twin births, and these in turn weighed more than those from triple parturitions; and, finally, (c) the mean birth weight obtained in experiment 3 was significantly higher than in other experiments.

The ADG of the kids, from birth to 9 kg live weight, was significantly affected by the following factors: genotype (*p* < 0.001), sex (*p* < 0.001), litter size (*p* < 0.001), experiment (*p* < 0.001) and genotype × litter size (*p* = 0.022). The rest of the factors and interactions considered in the model were not significant (*p* > 0.05). On average, BO × MG crossbred kids had a significantly higher ADG than MG kids (*p* < 0.001; Table 6) and it was observed in both male and female kids, and in the three types of births: single, double and triple. The average ADG increase in crossbred kids was 32% (Table 6). The following results were obtained with respect to the other factors that significantly affected the ADG (Table 6): (a) male kids had a significantly higher growth rate than females; (b) the ADG average obtained in experiment 1 was significantly higher than other experiments; furthermore, ADG of experiment 3 was higher than experiments 2 and 4; and, finally, (c) single birth kids grew faster than double birth kids and these in turn more than those of triple birth; however, these differences did not reach significance with purebred MG kids, which could explain the interaction genotype × litter size (Table 8).

The age of kids at 9 kg live weight variable was significantly affected by the following factors: genotype (*p* < 0.001), sex (*p* < 0.001), litter size (*p* < 0.001) and experiment (*p* < 0.001). For this variable, the remaining factors and all interactions considered in the model were not significant (*p* > 0.05). BO × MG crossbred kids reached 9 kg live weight earlier than MG kids (average of 19 d; Table 6) and this fact was observed in both male kids and female kids, and in single, twin and triple births. Regarding the other factors that significantly affected the age at 9 kg live weight variable, the following results were obtained (Table 6): (a) the male kids reached 9 kg live weight nine days earlier than the female kids; (b) single birth kids reached 9 kg live weight earlier than twin birth kids, and these in turn before the triple birth kids; and, finally, (c) in experiment 1 the kids reached 9 kg live weight at an age significantly earlier than in experiment 3, and these kids in turn before the kids of experiments 2 and 4.

The stillborn kids rate was not affected by any of the factors studied. Thus, in all of the experiments, this mortality was similar in the crossbred and MG purebred kids (8.0% and 8.4% of births, respectively). In contrast, mortality of kids during rearing was lower in crossbred kids than in MG kids (8.5% vs. 17.9%, respectively; OR = 0.47; *p* < 0.05). The same was found when total mortality (parturition plus rearing) of the kids was considered (16.5% vs. 26.3%; OR = 0.60; *p* < 0.05), so that in the crossbred kids, mortality decreased by 37% compared to the purebred kids. The other factors considered in the statistical model were not significant (*p* > 0.05).

### 3.3. Feed Conversion Rate of Milk Replacer

In experiment 1, the powdered milk consumption and milk replacer conversion rate variables were significantly affected by the factors genotype and sex, while the genotype × sex interaction was not significant (*p* > 0.05). BO × MG kids, relative to MG kids, consumed less milk powder (*p* < 0.001; Table 9) and had a more favourable conversion rate (1.16 ± 0.02 vs. 1.36 ± 0.02 kg DM/kg live weight gain; *p* < 0.001; Table 9). If we express the milk consumption in kg of commercial milk replacer (about 96% DM) we can estimate a conversion ratio of 1.21 kg/kg for BO × MG kids and 1.42 kg/kg for MG purebred kids. On the other hand, male kids, compared to female kids, also showed a lower consumption of powdered milk (*p* < 0.001) and a better conversion rate (*p* < 0.001; Table 9). In experiment 3 again, a better milk replacer conversion rate was obtained in the BO × MG kids (1.18 kg/kg live weight gain) than in MG kids (1.45 kg/kg live weight gain). In experiments 2 and 4, these data were not available due to accidents in the automatic feeders that made it impossible to record the actual consumption of powdered milk. The average milk replacer conversion rate for experiments 1 and 3 were 1.20 kg/kg for crossed kids and 1.43 kg/kg for MG purebred kids.

## 4. Discussion

In this work, it has been shown that the use of Boer bucks instead of MG bucks for the mating of Murciano-Granadina goats causes a decrease in the fertility (pregnancy rate: −8.1 percentage points; kidding rate: −13.7 percentage points) and prolificacy (−0.2 kids/parturition) of the does. In addition, the greatest differences occurred when mating was carried out during seasonal anoestrus (experiments 1, 2 and 4). In breeding season, other authors [26] did not find that the use of Boer bucks affected the fertility or prolificacy of a local breed (Spanish). However, we do not have any similar available work carried out in non-breeding season. On the other hand, the average kidding rate for MG goats (mated with MG bucks) in anoestrus season obtained in this work (78.62%) was similar to that obtained by [27] (75%) and greater than that obtained by [28] (46.3%).

However, we must point out that in experiment 4 the worst reproductive results were obtained, both with Boer bucks and MG bucks, but the cause is not clear. For example, in experiments 1 and 2, both also in the non-breeding season, the buck:doe ratio was similar to experiment 4, but fertility was higher.

In the temperate latitudes of the southern Iberian Peninsula, MG bucks are characterised by a high libido, even in the spring months [29], and in non-breeding season the MG does are completely responsive to the male effect [4]. In this regard, we can highlight that in our experiments it was observed that Boer males, mostly of German origin, showed a lower libido and interest in MG females than MG males did, especially in anoestrus season. This suggests that at the latitude at which the experiments were carried out (38–39° N), Boer males have lower sexual activity in non-breeding season and would have a lower capacity for stimulating the ovulatory process in MG goats. This hypothesis could explain, at least in part, the lower fertility of MG does mated to Boer bucks in the three experiments conducted in non-breeding season. A second hypothesis would be that Boer males suffer a greater deterioration of semen quality in non-breeding season. In MG bucks, some authors [30,31] have found that the semen quality of MG males shows a seasonal variation, but even in non-breeding season it is of sufficient quality to guarantee adequate fertility on the farm. In South Africa, the libido and semen quality of Boer bucks also varies seasonally [12,32]. However, at our latitude (Mediterranean area), there is no information available on the seasonal variation of semen quality of Boer bucks.

The lower prolificacy obtained with Boer males, compared to MG males, was due to a decrease in births with three and more offspring (14.7 vs. 22.4%), and an increase in births with one offspring (30.3 vs. 20.9%), the parturitions with twin births being similar (55.0 vs. 56.7%). We should point out that this result is not entirely negative, as MG breeders do not usually want their goats to have a high proportion of births with triplets, and especially quadruplets. In these cases, it is more common for goats to exhibit metabolic disease (pregnancy toxaemia) and increased mortality in pre-partum [33,34]. In addition, the MG kids are born with an excessively low birth weight, which generally leads to poorer adaptation to artificial rearing, slower growth rate [35] and more disorders and mortality during rearing [36].

In any case, the fact that Boer males showed poorer reproductive results in anoestrous season, compared to MG males, should not be a serious drawback, as they could probably be improved by applying stimulation treatments prior to mating, such as artificial manipulation of the photoperiod [37,38] or melatonin implants [28,38]. Nevertheless, this hypothesis should be confirmed in new studies.

In the four experiments carried out, the advantages of crossbred kids over pure MGs became evident. First, both the birth weight and the ADG of crossbred kids were higher than those of pure MG kids, and this was observed in both males and females and in the three types of parturition (single, double and triple) studied. The increase in birth weight and/or in ADG in the crossbred kids has also been observed in other works in which terminal crossbreeding of Boer bucks was performed in goats of local breeds of sizes similar to the MG breed (females of 30–50 kg adult weight: Spanish in USA [15,16], local breed from Mexico [17], White Improved in Poland [18], local breed from Brazil [19], Feral in Australia [39]). In contrast, the crossbreeding of Boer bucks with goats of larger adult size, such as Alpine or Saanen, scarcely improved the ADG of the crossbred kids [40,41]. On the other hand, the average birth weight obtained in this work in the MG purebred kids (2.36 and 2.14 kg for males and females) is similar to that cited by other authors in this same breed (ranging from 2.03 to 2.8 kg [6,7,8,42,43,44,45]). Likewise, the average ADG value (113 g/day) of the pure MG kids in artificial rearing falls within the range 96–125 g/d described in other works [7,8,22,46], but is lower than that cited by [6] (132 g/d), [42] (159 g/d) and [43] (153 g/d). Another interesting aspect of our study is that the ADG (both crossbred and purebred kids) in experiment 1 was higher than in other experiments. This result could have been, at least in part, because in experiment 1 the rearing system used (pens with two or three kids, artificial rearing in buckets, different commercial milk replacer) was also different from the other experiments.

Secondly, we can point out that the operators in charge of artificial rearing expressed their preference for crossbred kids, as they usually had to spend less time training them to drink milk from the teats in their first days of life. In addition, the mortality rate in artificial rearing for crossed kids was lower than that of the MG purebred kids. This last result could be explained, at least partially, by the higher birth weight of the crossbred kids and the lower frequency of triple and quadruple births, as in general, mortality is higher in kids with low birth weight [44,47].

Finally, in crossbred kids, the milk replacer conversion rate improved by 16% (average experiments 1 and 3), which can be explained by the fact that these kids also showed a higher growth rate. This relationship between growth rate and feeding efficiency was also observed by [15] in post-weaning growth of kids. Other authors [16] also found that the Boer × Spanish cross kids exhibited superior feed efficiency (13%) during the pre-weaning period compared to Spanish kids. Based on the average data obtained in this work, we can estimate that a BO × MG crossed kid consumes 7.45 kg of powdered milk from birth to 9 kg ((9–birth weight) × conversion rate = (9–2.79) × 1.20), while a purebred MG kid consumes 9.72 kg ((9–2.25) × 1.43). Therefore, crossbred kids consume 2.20 kg less milk powder, which means a saving of around 6.16 euros per kid (2.80 euros/kg milk replacer).

## 5. Conclusions

Carrying out terminal crossbreeding of Boer bucks with MG does (those not used to produce replacement kids) could be an interesting option for farmers of the latter breed, as crossbred kids have a higher birth weight (+24%) and, in artificial rearing, have lower mortality (−37%), a higher growth rate (+32%) and a better milk powder conversion rate (16%). However, when this crossbreeding is carried out in non-breeding season (experiments conducted at latitude 38–39° N), the reproductive performance of Murciano-Granadina goats worsens. In future work, it should be studied whether this problem can be avoided by applying a stimulation treatment to Boer males prior to mating.

## Figures and Tables

**Table 1:** Number (n) and live weight (LW, kg; mean ± SD) of bucks and goats, and number of kids used in each of four experiments performed to study the terminal crossbreeding of Boer (BO) bucks with Murciano-Granadina (MG) goats.

	Boer Bucks ^1^		MG Bucks ^1^		MG Goats		Kids (n) ^2^	
Experiment	n	LW	n	LW	n	LW	BO × MG	MG × MG
1	3	70 ± 2.5	3	53 ± 10.5	54	42 ± 8.9	26/24	45/33
2	4	77 ± 5.7	4	52 ± 11.9	92	41 ± 10.1	67/44	80/44
3	4	69 ± 3.8	4	44 ± 5.1	124	39 ± 10.9	77/57	88/72
4	4	72 ± 6.7	4	52 ± 7.0	84	40 ± 11.9	27/20	44/20
All	12	72 ± 5.5	12	59 ± 9.4	354	40 ± 10.7	197/145	257/169

^1^ Three Boer bucks were used in two different experiments. Three MG bucks were used in two different experiments. ^2^ Number of kids used for: birth weight study/growth rate study. Kids from parturitions with 4 and more kids/litter were not used in either of these studies.

**Table 2:** Number of bucks/number of goats (nulliparous or lactating) used in each mating group (groups 1 to 4) for each experiment performed to study the terminal crossbreeding of Boer (BO) bucks with Murciano-Granadina (MG) goats.

	Nulliparous Goats		Lactating Goats	
	Boer Bucks	MG Bucks	Boer Bucks	MG Bucks
Experiment ^1^	(Group 1)	(Group 2)	(Group 3)	(Group 4)
1	-	-	3/27	3/27
2	1/14	1/14	3/32	3/32
3	2/24	2/24	2/38	2/38
4	1/15	1/15	3/27	3/27

^1^ Nulliparous goats were not available in experiment 1.

**Table 3:** Means and odds ratios of the factors included in the final logistic regression model for pregnancy rate in Murciano-Granadina (MG) goats (primiparous and multiparous) mated with Boer bucks (n = 12) or MG bucks (n = 12) in 4 experiments.

Factors		n	Pregnancy Rate (%)	Odds Ratio	95% C.I. ^1^	*p*-Value
Buck breed	MG	172	86.6%	1.81	1.01–3.25	0.046
	Boer	172	78.5%			
Parity	1	104	89.4%	2.31	1.11–4.83	0.025
	≥2	240	79.6%			
Experiment	1	54	81.5%	2.36	0.99–5.67	0.054
	2	91	86.8%	2.88	1.30–6.36	0.009
	3	120	87.5%	2.87	1.37–6.01	0.005
	4	79	70.9%			

^1^ 95% Confidence Interval of Odds Ratio.

**Table 4:** Means and odds ratios of the factors included in the final logistic regression model for kidding rate in Murciano-Granadina (MG) goats (primiparous and multiparous) mated with Boer bucks (n = 12) or MG bucks (n = 12) in 4 experiments.

Factors		N	Kidding Rate (%)	Odds Ratio	95% C.I. ^1^	*p*-Value
Buck breed	MG	177	75.7%	1.99	1.24–3.21	0.005
	Boer	177	62.0%			
Parity	1	104	79.8%	2.68	1.51–4.78	0.001
	≥2	250	64.4%			
Experiment	1	54	75.9%	3.66	1.66–8.08	0.001
	2	92	77.2%	3.07	1.57–6.01	0.001
	3	124	70.1%	1.86	1.02–3.37	0.041
	4	84	54.8%			

^1^ 95% Confidence Interval of Odds Ratio.

**Table 5:** Means and odds ratios of the factors included in the final logistic regression model for prolificacy (kids born/parturition) in Murciano-Granadina (MG) goats (primiparous and multiparous) mated with Boer bucks (n = 12) or MG bucks (n = 12) in 4 experiments.

			Prolificacy ^1^						
Factors		n	1	2	≥3	m ± SD	Odds Ratio	95% C.I. ^2^	*p*-Value
Buck breed	MG	134	20.9	56.7	22.4	2.1 ± 0.75	1.98	1.18–3.31	0.009
	Boer	109	30.3	55.0	14.7	1.9 ± 0.68			
Parity	1	83	37.4	59.0	3.6	1.7 ± 0.55			
	≥2	160	18.7	54.4	26.9	2.1 ± 0.76	6.55	3.49–12.31	<0.001
Experiment	1	41	31.7	61.0	7.3	1.8 ± 0.58	0.63	0.26–1.53	0.31
	2	71	16.9	57.8	25.3	2.1 ± 0.71	3.83	1.76–8.31	<0.001
	3	85	20.0	55.3	24.7	2.1 ± 0.72	4.75	2.23–10.15	<0.001
	4	46	41.3	50.0	8.7	1.7 ± 0.78			

^1^ Parturition frequency (%) with 1, 2 or ≥3 kids/parturition; m ± SD = mean ± Standard Deviation of number of kids/parturition; n = number of parturitions. ^2^ 95% Confidence Interval of Odds Ratio.

**Table 6:** Least Square Means (LSM ± SE) of the birth weight and average daily gain (ADG) and age (AGE) from birth to 9 kg live weight of kids according to the genotype (crossbred and purebred), sex, litter size and experiment. Crossbred kids were from Boer bucks × Murciano-Granadina goats (BO × MG). Purebred kids were Murciano-Granadina breed (MG).

Factors		Birth Weight (kg)		ADG (g/d)		AGE (Days)	
		n	LSM ± SE	n	LSM ± SE	n	LSM ± SE
Genotype	MG	257	2.25 ± 0.04	169	114 ± 2.1	169	62 ± 1.1
	BO × MG	197	2.79 ± 0.05	145	150 ± 2.6	145	43 ± 1.3
	*p*-value	-	<0.001	-	<0.001	-	<0.001
Sex	Males	235	2.68 ± 0.04	167	140 ± 2.1	167	48 ± 1.1
	Females	219	2.36 ± 0.04	147	124 ± 2.2	147	57 ± 1.1
	*p*-value	-	<0.001	-	<0.001	-	<0.001
Litter size	Single	63	2.87 ± 0.06 ^a^	53	142 ± 2.9 ^a^	53	46 ± 1.5 ^c^
	Twin	278	2.45 ± 0.03 ^b^	188	132 ± 1.8 ^b^	188	53 ± 0.9 ^b^
	Triplets	113	2.24 ± 0.06 ^c^	73	122 ± 3.1 ^c^	73	59 ± 1.6 ^a^
	*p*-value	-	<0.001	-	<0.001	-	<0.001
Experiment	1	71	2.43 ± 0.07 ^b^	57	151 ± 3.4 ^a^	57	46 ± 1.7 ^c^
	2	147	2.44 ± 0.04 ^b^	88	123 ± 2.5 ^c^	88	56 ± 1.3 ^a^
	3	165	2.69 ± 0.04 ^a^	129	132 ± 2.0 ^b^	129	51 ± 1.0 ^b^
	4	71	2.52 ± 0.06 ^b^	40	122 ± 3.4 ^c^	40	57 ± 1.8 ^a^
	*p*-value	-	<0.001	-	<0.001	-	<0.001

^a,b,c^ For each factor, means values within a column with unlike superscript letters were significantly different at *p* < 0.05.

**Table 7:** Least Square Means (LSM ± SE) of the birth weight (kg) of kids according to genotype (crossbred and purebred) × sex interaction. Crossbred kids were from Boer bucks × Murciano-Granadina goats (BO × MG). Purebred kids were Murciano-Granadina breed (MG).

Genotype	Sex			
	Males		Females	
	n	LSM ± SE	n	LSM ± SE
MG	126	2.36 ± 0.05 ^c^	131	2.14 ± 0.05 ^d^
BO × MG	109	3.00 ± 0.06 ^a^	88	2.58 ± 0.06 ^b^

^a,b,c,d^ Means values with unlike superscript letters were significantly different at *p* < 0.01.

**Table 8:** Least Square Means (LSM ± SE) of the average daily gain (ADG, g/d) from birth to 9 kg live weight of kids according to the genotype (crossbred and purebred) × litter size interaction. Crossbred kids were from Boer bucks × Murciano-Granadina goats (BO × MG). Purebred kids were Murciano-Granadina breed (MG).

Genotype	Litter Size					
	Single		Twins		Triplets	
	n	LSM ± SE	n	LSM ± SE	n	LSM ± SE
MG	28	119 ± 4.0 ^d^	96	112 ± 2.4 ^d^	45	109 ± 3.7 ^d^
BO × MG	25	166 ± 4.3 ^a^	92	152 ± 2.6 ^b^	28	134 ± 5.0 ^c^

^a,b,c,d^ Means values with unlike superscript letters were significantly different at *p* < 0.05.

**Table 9:** Least Square Means (±SE) of initial and final live weights and consumption and feed conversion rate for milk replacer of kids according to the genotype (crossbred and purebred) and sex. Crossbred kids were from Boer bucks × Murciano-Granadina goats (BO × MG). Purebred kids were Murciano-Granadina breed (MG).

		Genotype			Sex	
Variable	MG	BO × MG	*p*-Value	Males	Females	*p*-Value
Number of kids	23	23	-	23	23	-
Number of pens	8	9	-	9	8	-
Starting live weight/pen/kid (kg)	2.87 ± 0.09	3.12 ± 0.09	0.077	3.35 ± 0.09	2.64 ± 0.09	<0.001
Final live weight/pen/kid (kg)	9.32 ± 0.10	9.46 ± 0.10	0.352	9.48 ± 0.10	9.31 ± 0.10	0.255
Duration/pen/kid (d)	46 ± 1.8	33 ± 1.7	<0.001	35 ± 1.7	43 ± 1.8	0.010
Milk replacer/pen/kid (kg DM)	8.7 ± 0.19	7.4 ± 0.18	<0.001	7.5 ± 0.18	8.6 ± 0.19	<0.001
Feed conversion rate ^1^ (kg DM/kg)	1.36 ± 0.02	1.16 ± 0.02	<0.001	1.20 ± 0.02	1.30 ± 0.02	0.001

^1^ kg DM milk replacer/kg kids live weight gain.

## Data Availability

The data presented in this study are available on request from the corresponding author.
